# DisGeNET: a discovery platform for the dynamical exploration of human diseases and their genes

**DOI:** 10.1093/database/bav028

**Published:** 2015-04-15

**Authors:** Janet Piñero, Núria Queralt-Rosinach, Àlex Bravo, Jordi Deu-Pons, Anna Bauer-Mehren, Martin Baron, Ferran Sanz, Laura I. Furlong

**Affiliations:** ^1^Research Programme on Biomedical Informatics (GRIB), Hospital del Mar Medical Research Institute (IMIM), Department of Experimental and Health Sciences, Universitat Pompeu Fabra, C/Dr Aiguader 88, E-08003 Barcelona, Spain, ^2^Roche Pharma Research and Early Development, pRED Informatics, Roche Innovation Center Penzberg, Roche Diagnostics GmbH, Nonnenwald 2, 82377 Penzberg, Germany and ^3^Scientific & Business Information Services, Roche Diagnostics GmbH, Nonnenwald 2, 82377 Penzberg, Germany

## Abstract

DisGeNET is a comprehensive discovery platform designed to address a variety of questions concerning the genetic underpinning of human diseases. DisGeNET contains over 380 000 associations between >16 000 genes and 13 000 diseases, which makes it one of the largest repositories currently available of its kind. DisGeNET integrates expert-curated databases with text-mined data, covers information on Mendelian and complex diseases, and includes data from animal disease models. It features a score based on the supporting evidence to prioritize gene-disease associations. It is an open access resource available through a web interface, a Cytoscape plugin and as a Semantic Web resource. The web interface supports user-friendly data exploration and navigation. DisGeNET data can also be analysed via the DisGeNET Cytoscape plugin, and enriched with the annotations of other plugins of this popular network analysis software suite. Finally, the information contained in DisGeNET can be expanded and complemented using Semantic Web technologies and linked to a variety of resources already present in the Linked Data cloud. Hence, DisGeNET offers one of the most comprehensive collections of human gene-disease associations and a valuable set of tools for investigating the molecular mechanisms underlying diseases of genetic origin, designed to fulfill the needs of different user profiles, including bioinformaticians, biologists and health-care practitioners. **Database URL: **http://www.disgenet.org/

## Background

Biomedical sciences are facing an enormous increase of data available in public sources, not only in volume, but also in nature (the so-called *Biomedical Big Data*). Translational bioinformatics has emerged as a new field to transform the huge wealth of biomedical data into clinical actions using bioinformatic approaches (1). By the integrative exploitation of genomic, phenomic and environmental information, translational bioinformatics will enable a deeper understanding of disease mechanisms (2). In the pursuit to implement personalized medicine, the clinical practitioners will increasingly rely on informatic resources that aid in the exploration and interpretation of data on the genetic determinants of disease (3). The availability of both, comprehensive knowledge sources on disease genes and tools that allow their analysis and exploitation, should lay the basis to achieve this goal. Currently, there are several resources that cover different aspects of our current knowledge on the genetic basis of human diseases (4–11). DisGeNET is one of these resources (12, 13), whose aims are to cover all disease areas (Mendelian, complex and environmental diseases), with special care on the integration and standardization of data, and to provide open access on knowledge of genes associated to human diseases.

**Figure 1.**
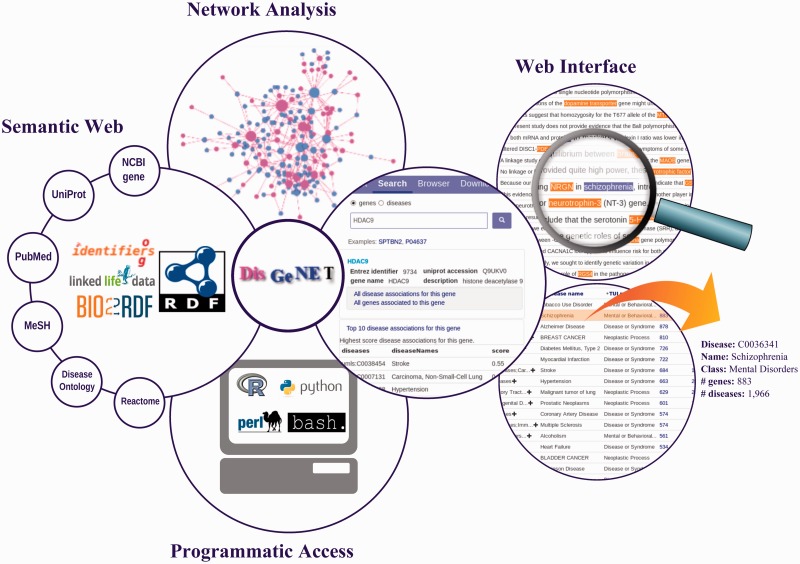
The main features of the DisGeNET discovery platform. DisGeNET is available through a web interface, a Cytoscape plugin, as a Semantic Web resource, and supports programmatic access to its data.

In this article, we present the ‘DisGeNET discovery platform’, which includes a new version of the database and, more importantly, a new set of ‘analysis tools’ to facilitate and foster the study of the molecular underpinning of human diseases (Figure 1). An important aspect of the DisGeNET toolkit is to support different types of users. Since the scientific literature represents a rich, up-to-date source of knowledge on disease genes, the database also includes gene-disease associations (GDAs) mined from MEDLINE via a NLP-based approach (14).

One of the key features of DisGeNET is the explicit representation of the provenance of the information, which allows the user to trace back to the original source of information and, more importantly, to explore the data in its original context. These aspects are of crucial importance to evaluate the evidence supporting a scientific assertion, in order to determine its relevance for translational applications. Moreover, the DisGeNET discovery platform allows prioritizing GDAs on the basis of the evidence supporting the data.

Semantic Web and Linked Data approaches have become increasingly important to life sciences and health care, since they properly meet the data standardization and integration requirements of translational biomedical research (15, 16). For this reason, the information contained in DisGeNET has been formally represented as Resource Description Framework (RDF) and linked to the Linking Open Data Cloud (http://lod-cloud.net/). The integration of DisGeNET in the emerging Semantic Web intends to ease and foster the integrated use of its data with other resources available in the web, and to support and expand research on human diseases and their genes.

The ‘DisGeNET discovery platform’ allows easy browsing and downloading of the information related to human diseases and their genes. The platform supports different types of users: the bioinformatician and software developer that interrogates the database by customized scripts or using Semantic Web technologies, the systems biology expert that explores and analyses the network representations of the information, and biologists and health-care practitioners who interrogate the database using its user-friendly web interface (Figure 1 and Box 1). Its comprehensiveness, standardization, availability and accessibility, as well as the suite of analysis tools and support of different user profiles make DisGeNET a resource of choice to investigate diseases of genetic origin.

**Box 1. The DisGeNET discovery platform**

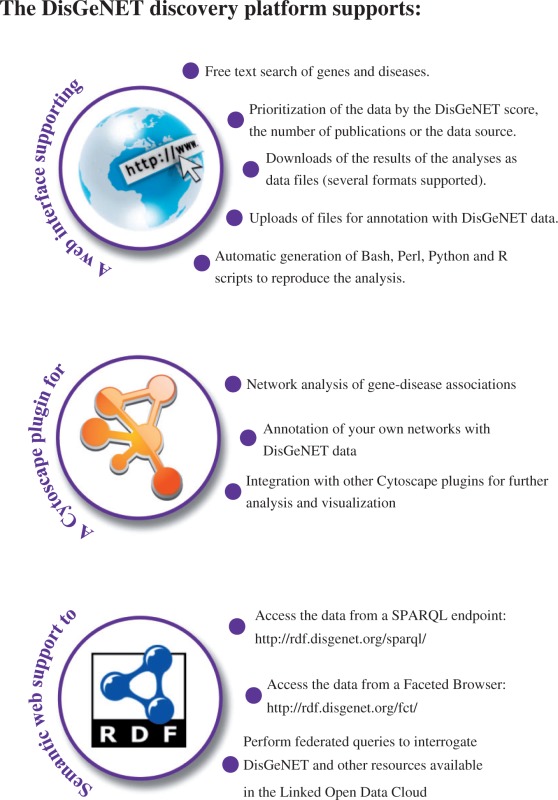

## Results

### DisGeNET database

#### Statistics

DisGeNET database (v2.1, release May, 2014) contains 381 056 GDAs between 16 666 genes and 13 172 diseases, representing one of the most comprehensive currently available resources on diseases and their genes. The DisGeNET resources are permanently updated. All DisGeNET data is freely available under an Open Database license model (for more details see http://www.disgenet.org/ds/DisGeNET/html/legal.html).

The information contained in DisGeNET is obtained from expert-curated databases, namely CTD (7), UniProt (17), Rat Genome Database (RGD) (18) and Mouse Genome Database (MGD) (19). It also contains data obtained from the scientific literature by different text mining approaches. These include data extracted from published peer-reviewed articles on Genome Wide Association Studies (GWAS) Genetics Association Database (GAD) (8), a literature-derived human gene-disease network (LHGDN) dataset, obtained by mining Entrez Gene’s GeneRIF database (20) using conditional random fields (21), and the BeFree dataset, composed of GDAs obtained from MEDLINE abstracts by a NLP-based approach (14).

DisGeNET data have been aggregated according to origin and level of curation into CURATED (expert-curated associations obtained from UniProt and CTD human datasets), PREDICTED (containing human GDAs inferred from mouse and rat models, i.e. CTD mouse and CTD rat datasets, and RGD and MGD datasets), and ALL. Table 1 displays the statistics of the current version of the database (v2.1) compared with the first published release (v1.0), showing a significant increase in terms of genes, diseases and their associations (2.2, 3.2 and 9.3 times increase, respectively). The newly added BeFree dataset contributes with 330 888 GDAs corresponding to 10 557 diseases and 13 402 genes, from which 3484 genes, 6354 diseases and 294 311 associations are not present in any other of the contributing sources. Although the information obtained from the CURATED sources is of high quality, the inherent paucity of manual curation of the literature demands to complement expert-curated associations with data automatically identified from the current literature. In this way, we keep up-to-date with the most recent findings, otherwise locked as unstructured data, and better reflect new avenues of research on the mechanisms of diseases in the database.

**Table 1.** Comparison of the DisGeNET current release (v2.1) with the first (v1.0) release.

Sources	Genes		Diseases		Associations	
	v1.0	v2.1	**v1.0**	**v2.1**	**v1.0**	**v2.1**
**UNIPROT**	1240	1839	1475	2376	1762	2622
**CTD human**	3345	6983	2702	4860	6853	21 925
**CURATED**	3820	7108	3096	5466	8261	22 678
**MGD**	0	1197	0	1059	0	1624
**RGD**	0	1392	0	737	0	6135
**CTD mouse**	0	51	0	40	0	52
**CTD rat**	0	11	0	10	0	11
**PREDICTED**	0	2272	0	1758	0	7800
**GAD**	0	9045	0	1737	0	33 940
**LHGDN**	6154	6136	1850	1846	34 552	34 487
**BeFree**	0	13 402	0	10 557	0	330 888
**ALL**	7314	16 666	4046	13 172	40 729	381 056

**Figure 2.**
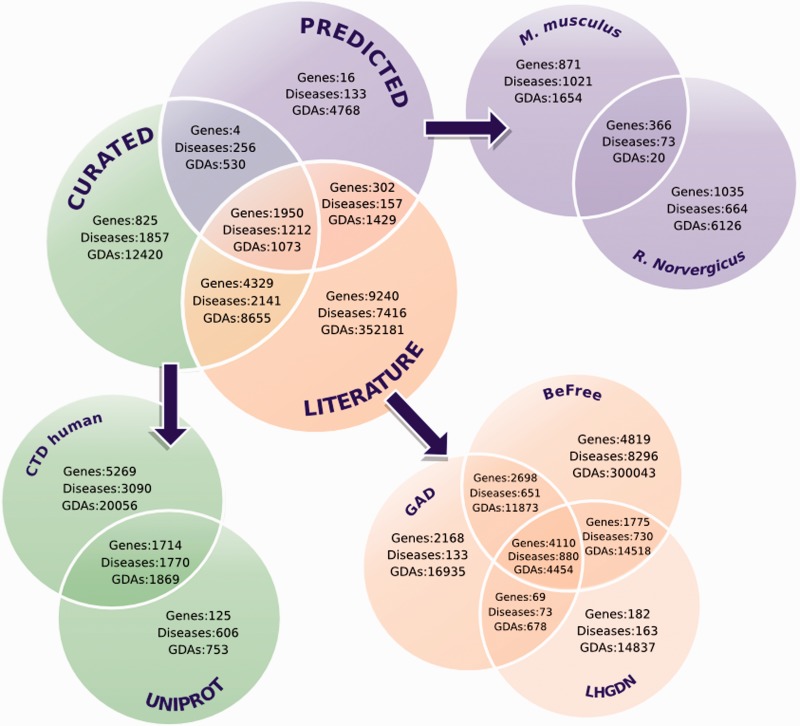
Venn diagrams showing the overlaps among genes, diseases and GDAs according to their source. LITERATURE corresponds to GAD, BeFree and LHGDN.

The overlap among the different data sources, for associations, genes and diseases is shown in Figure 2. Only 0.3% of the GDAs (1073 GDAs) are common to all the DisGeNET datasets, while in the case of genes and diseases the overlap is 12 and 9%, respectively. Moreover, if we focus on the CURATED dataset only, we find 71% of UniProt associations contained in the CTD human dataset. There is little overlap (<1%) between the animal models’ data, in terms of genes, diseases and associations. In the case of the text-mined data (LITERATURE in Figure 2), 48% of GAD and 55% of LHGDN are included in the BeFree associations’ dataset. As we previously reported (13), these small overlaps between different data sources evidence the existence of knowledge pockets in this field and highlight, still today, the pressing need for data integration.

#### Gene attributes

**Figure 3.**
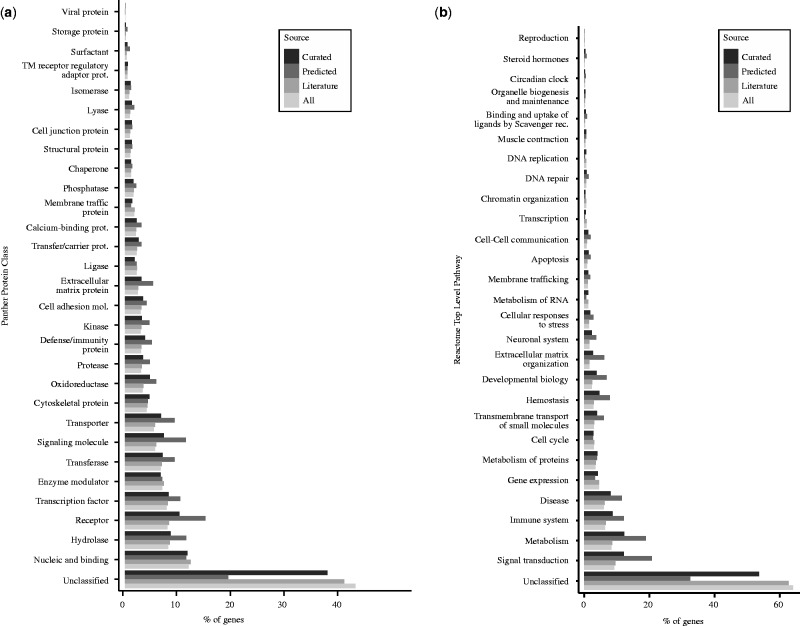
Distribution of DisGeNET genes by Panther protein class (a), and by Reactome pathways (b). Note that for both classifications, we used the top-level class.

We provide the official gene symbol from the HUGO Gene Nomenclature Committee (HGNC), the NCBI Official Full Name, and the annotation to proteins using the UniProt accession number. In addition, genes are classified according to the PANTHER Protein Class Ontology (22, 23) and Reactome top-level pathways (24) (Figure 3a and b, respectively).

More than 80% of DisGeNET genes encode proteins (nearly 14 000 genes), while the remaining genes are pseudogenes, ncRNA and other categories. Recent estimations indicate that the number of protein-coding genes in the human genome is ∼19 000 (25). Thus, DisGeNET includes annotations to diseases for about the 70% of human protein-coding genes. The largest protein class in DisGeNET is ‘nucleic acid-binding protein’, comprising 12% of all disease proteins (Figure 3a). The next best-represented categories are ‘hydrolases’, ‘receptors’ and ‘transcription factors’, comprising ∼8% each. Transporters, signaling proteins and transferases, are the next better-represented protein categories (Figure 3a). These results do not change significantly if we restrict the analysis to the CURATED dataset. Note that ∼40% of disease proteins in DisGeNET are not covered by this ontology (‘Unclassified’). The highest coverage in terms of protein class is for the PREDICTED dataset (only 19% of genes remain unclassified).

We also provide information on pathways in which genes participate using the Reactome database (24) (Figure 3b). The best-represented pathways are ‘Metabolism’ and ‘Signal Transduction’, comprising ∼10–20% of the disease proteins each. Nevertheless, more than half of DisGeNET proteins (64% of ALL and 54% of CURATED) are not covered by Reactome pathways (‘Unclassified’). This coverage is higher for the PREDICTED sources (68% genes are annotated to pathways). ‘Immune System’ and ‘Disease’ are the next best-represented top-level pathways, concentrating 6–12% of the genes in the different datasets.

#### Disease attributes

**Figure 4.**
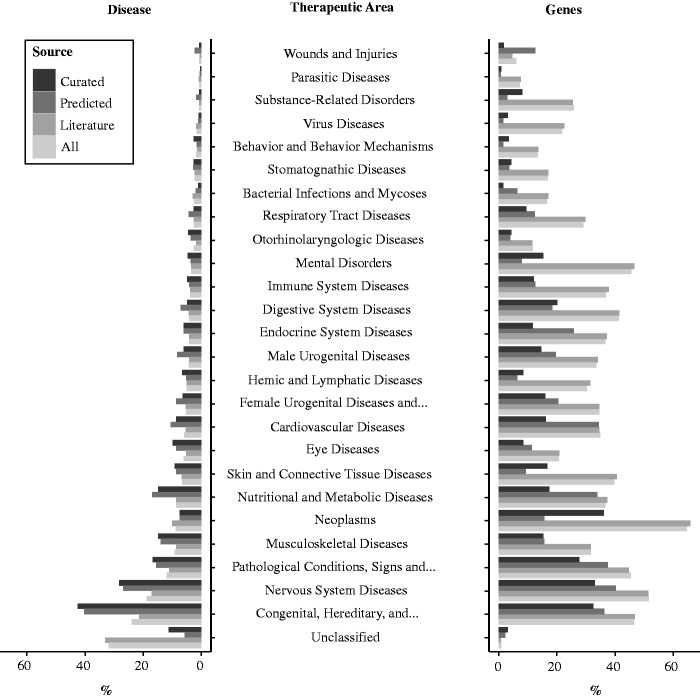
Distribution of diseases and genes according to the MeSH disease classification.

Diseases are annotated with the UMLS concept identifiers and semantic types, and classified according to the Medical Subject Headings classes (MeSH) hierarchy using the 23 upper level terms of the MeSH tree branch C (Diseases) plus three upper level terms of the F branch (Psychiatry and Psychology: ‘Behavior and Behavior Mechanisms’, ‘Psychological Phenomena and Processes’, and ‘Mental Disorders’). Diseases that could not be classified in the MeSH hierarchy were labeled as ‘Unclassified’ (Figure 4). The largest MeSH disease class in DisGeNET is ‘Congenital, Hereditary and Neonatal Diseases and Abnormalities’ (C16). Twenty four percent of DisGeNET diseases are annotated to this class, while 46% of the DisGeNET genes are associated to diseases annotated to this class (ALL dataset). The second more populated disease class is ‘Nervous System Diseases’ (19% diseases, ALL dataset), while approximately half of disease genes (51%, ALL dataset) are annotated to this class (Figure 4). The disease class ‘Neoplasms’ represents 9% of diseases (ALL dataset), whereas this category contains the largest number of DisGeNET genes (65% genes, ALL dataset). In addition, we also provide annotations to the Human Disease Ontology (HDO) (26), to the International Classification of Diseases, Version 9, Clinical Modification (ICD9-CM) (‘Classification of Diseases and Injuries’. Cdc.gov. National Center for Health Statistics, 2009), and to the Human Phenotype Ontology (HPO) (27) (only available in the RDF). Supplementary Table S1 shows the coverage of DisGeNET diseases with the different disease terminologies and ontologies.

**Figure 5.**
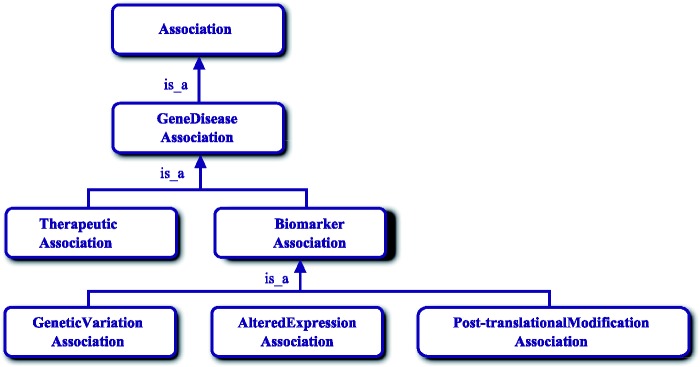
The DisGeNET association type ontology.

#### GDA attributes

The GDAs in DisGeNET are classified according to the association type using the ‘DisGeNET association type ontology’ (Figure 5), and are annotated with the DisGeNET score (explained in the next section) and the supporting evidence. The publications (PubMed identifiers, PMIDs) reporting the GDA, a representative sentence from each publication describing the association between the gene and the disease, and the source of provenance are provided as supporting evidence. For associations with more than 10 publications, we limit the information to the 10 most recent ones. If a representative sentence is not found, we provide the title of the article. Almost all (>99%) of the GDAs are supported by a publication. Remarkably, 67% of the articles supporting the GDAs in DisGeNET have been published in the last 10 years, and 37% in the last 5 years. Finally, if there is a genetic variant associated to the GDA, we provide its dbSNP identifier.

### The DisGeNET score: ranking GDAs

One of the main problems of exploiting large collections of aggregated biomedical data is how to prioritize the information. The current release of DisGeNET contains over 300 000 GDAs integrated from different sources. To help users to prioritize and select GDAs in DisGeNET, a score for each GDA based on the supporting evidence has been implemented. The DisGeNET score takes into account the number of sources that report the association, the type of curation of each of these sources, the animal models where the association has been studied, and the number of supporting publications from text-mining based sources. The score ranges from 0 to 1 and is computed according to the formula described in ‘Methods’ section. The DisGeNET score allows obtaining a ranking of GDAs and a straightforward classification of curated vs predicted vs literature-based associations since it stratifies the associations based on their level of evidence. For instance, associations only reported by UniProt or CTD, which have been curated by experts, have higher scores (i.e. associations with *S* ≥ 0.3) than those only supported by animal models or text-mining based sources. For a more detailed guide to the possible DisGeNET score values according to the supporting evidence see http://www.disgenet.org/web/DisGeNET/menu/dbinfo#score.

The top-20 scoring GDAs from DisGeNET (Table 2) are very well-studied gene-disease relationships, like Alzheimer Disease and APP [amyloid beta (A4) precursor protein], obesity and MC4R (melanocortin 4 receptor), and Type 2 Diabetes Mellitus and IRS1 (insulin receptor substrate 1). For many cases, the genes have been named after the disease, or have the name of the disease as a synonym (ATP7B and Wilson Disease, see: http://www.genenames.org/cgi-bin/gene_symbol_report?hgnc_id=870, CFTR and cystic fibrosis, APP and Alzheimer Disease, see: http://www.genenames.org/cgi-bin/gene_symbol_report?hgnc_id =620, ASPA and Canavan Disease, http://www.genename s.org/cgi-bin/gen e_symbol_report?hgnc_id=756). The association between ATP7B gene (ATPase, Cu++ transporting, beta polypeptide) and Wilson’s disease (C0019202, Wilson’s Disease or Hepatolenticular Degeneration) is the one achieving the highest score in DisGeNET. Wilson’s disease is an autosomal recessive disease characterized by the deposition of copper in brain, liver, cornea, and other organs. From its initial description in 1912 (28), it has been shown to be related to mutations in the ATP7B gene (29), that encodes a copper transporter of the family of ATPases, leading to copper accumulation. Wilson’s disease is a very well-described Mendelian disorder with established animal models (30, 31), and therefore obtains the maximum DisGeNET score value. In summary, the DisGeNET score provides an intuitive, evidence-based way to rank and prioritize GDAs.

**Table 2.** Distribution of the score components for the 20 highest scored associations.

Disease	Gene	Score	UNIPROT	CTD human	Rat	Mouse	Number of articles		
							BeFree	GAD	LHGDN
Hepatolenticular Degeneration	ATP7B	0.9898	0.3	0.3	0.1	0.1	174	31	23
Obesity	MC4R	0.9400	0.3	0.3	0.1	0.1	220	46	0
Diabetes Mellitus, Type 2	IRS1	0.9077	0.3	0.3	0.1	0.1	93	33	0
Cystic Fibrosis	CFTR	0.9000	0.3	0.3	0	0.1	1429	150	78
Rett Syndrome	MECP2	0.9000	0.3	0.3	0	0.1	438	27	43
Alzheimer Disease	APP	0.8820	0.3	0.3	0	0.1	1096	18	81
Creutzfeldt-Jakob Syndrome	PRNP	0.8731	0.3	0.3	0	0.1	285	16	23
Familial Mediterranean Fever	MEFV	0.8702	0.3	0.3	0	0.1	282	48	12
Gastrointestinal Stromal Tumors	KIT	0.8648	0.3	0.3	0	0.1	526	13	31
Pheochromocytoma	RET	0.8644	0.3	0.3	0.1	0.1	142	2	6
Muscular Dystrophy, Duchenne	DMD	0.8542	0.3	0.3	0	0.1	670	12	21
Diabetes Insipidus, Neurogenic	AVP	0.8463	0.3	0.3	0.1	0.1	90	2	5
Fragile X Syndrome	FMR1	0.8451	0.3	0.3	0	0.1	505	13	16
Ornithine Carbamoyltransferase Deficiency Disease	OTC	0.8432	0.3	0.3	0.1	0.1	125	1	1
Brugada Syndrome	SCN5A	0.8419	0.3	0.3	0	0.1	166	10	23
Marfan Syndrome	FBN1	0.8414	0.3	0.3	0	0.1	287	9	20
Polycythemia Vera	JAK2	0.8406	0.3	0.3	0	0.1	286	6	39
Polycystic Kidney, Autosomal Recessive	PKHD1	0.8273	0.3	0.3	0.1	0.1	41	0	6
Malignant Hyperthermia	RYR1	0.8256	0.3	0.3	0	0.1	184	11	13
Canavan Disease	ASPA	0.8228	0.3	0.3	0.1	0.1	48	1	2

### The DisGeNET discovery platform

The ‘DisGeNET discovery platform’ is composed of a web interface, a Cytoscape plugin, a SPARQL endpoint and a Faceted Browser (Box 1). In addition, the DisGeNET data are available for downloading in several formats: as SQLite database, as tab-separated files and as dump files, serialized in RDF/Turtle.

#### Web interface

DisGeNET web interface is one of the main new features of the current release. It has been designed to make it easier to search, visualize, filter and share the data. In addition, it allows downloading data files containing the results of the user’s search in a variety of formats. Moreover, it automatically generates scripts in several programming languages that can be downloaded and used to reproduce the analyses performed by the user. Advanced users may customize these scripts to perform similar queries and/or incorporate them into their own bioinformatic workflows. Lastly, functionalities are offered to share the results of searches performed with DisGeNET via e-mail or by embedding the HTML code of the results page in a web page.

**Figure 6.**
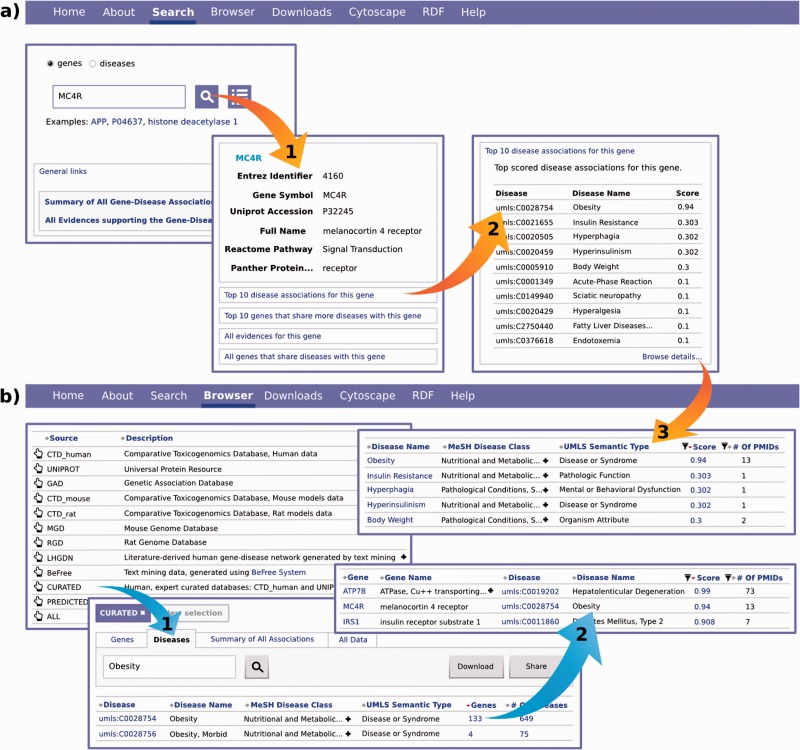
The two entry points to the web interface: the Search view **(a)** and the Browse view **(b)**.

There are two entry points to the web interface. The Search view (Figure 6a) and the Browse view (Figure 6b). The first allows the user to perform free-text searches on the database on a specific gene (or disease). As a result, the user retrieves all diseases (or genes) that are associated with this gene (or disease). Additionally, the user can perform queries to the database with a list of genes or diseases. The Browse view allows the user to explore all the information starting by a specific data source (e.g. CTD). In both cases, the results can be filtered by the DisGeNET score, data source and some of the attributes of the data, such as the PANTHER protein class, MeSH disease class or the DisGeNET association type class. The web interface also provides links to external resources such as NCBI Gene and UniProt for genes, Linked Life Data for diseases, dbSNP for genomic variants and PubMed for the original publications. The user can also inspect the evidences of a GDA, exploring the sentences extracted from the supporting publications, in which the gene, variants and disease are highlighted. Finally, the DisGeNET discovery tool also offers the exploration of the disease-centric and gene-centric views of the data, supporting the analysis of disease comorbidities and the study of common mechanisms of genes associated to shared diseases (32). For more details on the functionalities offered by the web interface, see the Web Interface user guide (http://www.disgenet.org/ds/DisGeNET/files/DisGeNET_webInterface_userGuide.pdf).

#### The DisGeNET Cytoscape plugin

The DisGeNET Cytoscape plugin allows to visualize, query and analyse a network representation of DisGeNET database (12, 13). The GDAs are represented as bipartite graphs, in which genes and diseases are the vertices and the associations are represented as edges. The data can also be analysed from a gene-centric or a disease-centric view, by using the network projections (gene–gene networks and disease–disease networks). In the network projections, nodes are connected if they share a neighbor in the original bipartite graph, and are particular useful as a representation of the diseasome, or for exploring group of genes related to common diseases.

The user can perform queries restricted to a gene, a disease, or to a particular source, DisGeNET association type class, or MeSH disease class. Moreover, the DisGeNET Cytoscape plugin allows the user to leverage the network visualization and analysis tools available in Cytoscape, and to seamless integrate DisGeNET functionalities with other Cytoscape plugins to perform network analysis, functional annotation enrichment, and many other analysis on DisGeNET networks. Finally, DisGeNET Cytoscape plugin allows the annotation of other types of networks (e.g. protein interaction networks, signaling or metabolic pathways, drug-disease networks) with gene-disease information.

#### DisGeNET in the Semantic Web

**Figure 7.**
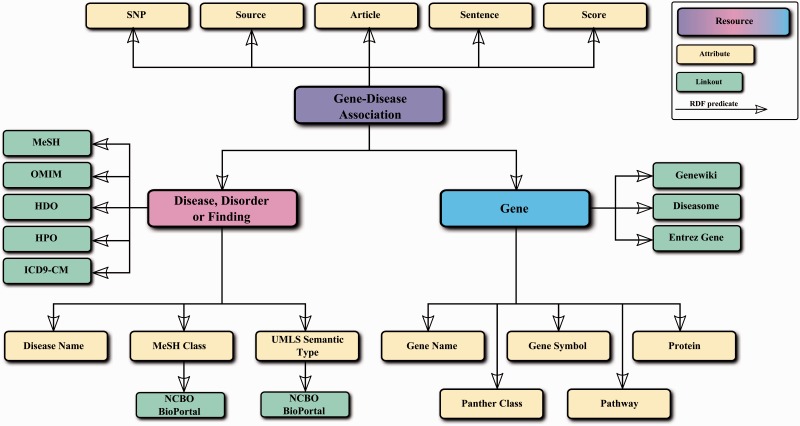
Simplified data model of the DisGeNET RDF representation.

DisGeNET data are also available as a RDF-linked dataset, to extend the Linked Data space with GDAs. The RDF version of DisGeNET is a set of triples centered on the GDA concept (see Figure 7), where information such as genetic variations or the pathways where disease genes are known to be involved, is linked in the DisGeNET RDF graph.

A faceted browser and a SPARQL endpoint have been implemented to access and navigate DisGeNET linked data. Importantly, the SPARQL endpoint supports query federation, which allows integrating DisGeNET GDAs with different types of data available in the Linked Data space. As an example of how making data available as Linked Data using Semantic Web technologies and open and standard ontologies promotes integration with other Linked Data sources, the RDF version of DisGeNET has been integrated in the Open PHACTS Discovery Platform, which is the goal product of the Open PHACTS project (33). The RDF schema, the data dump, the Vocabulary for Interlinked Dataset (VoID) description file, the faceted browser and the SPARQL endpoint can be accessed in the RDF section of the DisGeNET discovery platform (http://rdf.disgenet.org/).

#### Use case

**Figure 8.**
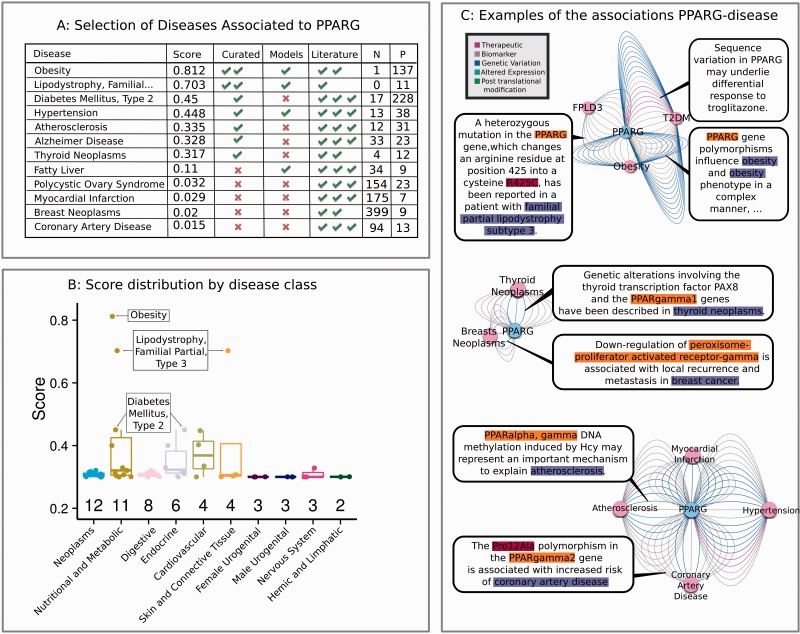
Highlights of the information that can be extracted from DisGeNET, using PPARG as example. **(a)** Selection of the diseases associated to PPARG, with the number of data sources supporting them. N: Number of genes annotated to the disease with score higher than or equal to PPARG. P: Number of articles supporting the association. **(b)** Distribution of scores by disease class, for the 42 diseases reported by curated sources. Only classes with more than one disease are shown. The number of disease terms in each class is shown on the top of the x-axis. **(c)** Examples of PPARG relations to a selection of diseases. The networks were obtained with the DisGeNET Cytoscape plugin. The colors of edges reflect different association types.

We illustrate the use of the DisGeNET platform with the example of the peroxisome proliferator-activated receptor gamma (PPARG) gene. PPARG is a ligand-activated transcription factor, abundant in adipose tissue, where it is involved in the regulation of transcription of genes related to adipogenesis and glucose and lipid metabolism (34). DisGeNET associates PPARG to >300 diseases, although only two of them, Obesity and Lipodystrophy, Familial Partial, Type 3 (FPLD3), stem from two expert-curated resources, UniProt and CTD (Figure 8a). DisGeNET also includes data on several mouse and rat disease models where alterations in this gene have been described. Interestingly, 281 disease associations are not reported by any of the curated resources, and are captured only by text mining the scientific literature. Figure 8a shows a representative set of the top and bottom-scoring disease phenotypes associated to PPARG, and the number of original sources that report them.

DisGeNET also allows filtering PPARG-associated diseases by their MeSH classes, presenting a global picture of the involvement of PPARG gene in human diseases. Figure 8b shows a plot with the distribution of DisGeNET scores of PPARG-associated diseases from curated sources by MeSH class. The two best-represented disease classes are Neoplasms (12 diseases), and Nutritional and Metabolic Diseases (11 diseases). The latter category includes the top three highest scoring diseases, in agreement with the role of PPARG in nutrient levels sensing and the modulation of lipid and glucose metabolism (34).

Obesity is the top ranking disease associated to PPARG (0.812). This association is supported by almost all sources, and by >100 articles (Figure 8a), which mostly explore the role of PPARG genetic variants in Obesity (35–38). This is illustrated in Figure 8c: many of the edges connecting PPARG and Obesity correspond to the ‘Genetic Variation’ Association Type.

The second highest ranking (0.703) disease association in Figure 8a, FPLD3, caused by germline heterozygous inactivating mutations in PPARG gene (39). An illustrative sentence of the PPARG-FPLD3 relationship is shown in Figure 8c (40).

The third highest scoring association is PPARG-Type 2 Diabetes Mellitus (T2DM, 0.45), supported by a wealth of literature (228 articles in DisGeNET). By exploring the evidences (Figure 8c), therapeutic relations between PPARG and troglitazone stand out (41–43). Many of the other evidences are reports of GWAS studies, linking T2DM and the effect of several genetic variants (blue edges in Figure 8c).

## Methods

### Data source extraction

UniProt: UniProt/SwissProt is a database containing curated information about protein sequence, structure and function (17). Disease associated proteins were obtained from the ‘humsavar’ file, along with the dbSNP identifier(s) associated to the disease (Supplementary Table S2 lists all the files used for database development indicating name of the file, URL and date of accession). For these proteins, we also downloaded the full records, from where we extracted information on the PMID supporting the association. UniProt GDAs were assigned to the type ‘Genetic Variation’ from the ‘DisGeNET association type ontology’. UniProt provided 2,622 GDAs between 1,839 genes and 2,376 diseases.

The Comparative Toxicogenomics Database is aimed at understanding the effects of environmental chemicals on human health, and contains expert curated information on gene-disease relationships (7). We obtained direct GDAs (excluding the ones mediated by chemicals) and PMIDs for human, mouse, and rat. GDAs obtained from CTD are classified as ‘Biomarker’ or ‘Therapeutic’ classes from the DisGeNET association type ontology, according to its labeling in the original source (‘Marker’ or ‘Therapeutic’). CTD provided 21 925 associations for 4860 diseases and 6983 genes.

The MGD is the international community resource for integrated genetic, genomic and biological data obtained using mouse as animal model (19). MGD provides full annotation of phenotypes and disease associations for mouse models (genotypes) using terms from the Mammalian Phenotype Ontology and disease names from OMIM. GDAs obtained from MGD are assigned to the association type class ‘Biomarker’ from the DisGeNET association type ontology. MGD provided 1624 associations between 1197 genes and 1059 diseases.

The RGD is a collaborative effort between leading research institutions involved in rat genetic and genomic research (18). We obtained GDAs and PMIDs for rat models of disease and the information of human orthology. We did not include the associations labeled as ‘resistance’, ‘induced’ or ‘no association’, nor the ones annotated with the following evidence codes ‘Inferred from electronic annotation’, ‘Inferred from sequence or structural similarity’ and ‘Non-traceable author statement’. GDAs obtained from RGD assigned to the association type class ‘Biomarker’ from the DisGeNET association type ontology, except for those labeled as ‘treatment’, which are classified as ‘Therapeutic’. RGD provided 6135 associations between 1392 genes and 737 diseases.

The GAD is an archive of human genetic association studies of complex diseases. GAD is primarily focused on archiving information on complex human diseases (8). It includes a curated summary extracted from articles on candidate gene and GWAS published in peer-reviewed journals. We extracted GDAs that were supported by publications and were not labeled as ‘negative’ or as ‘normal variation’. We kept information on the PMID, the dbSNP identifier annotated to the association, and the title or conclusion of the study when provided. GDAs obtained from GAD are assigned the association type ‘Genetic Variation’ from the DisGeNET association type ontology. The DisGeNET GAD dataset contains 33 940 associations among 9045 genes and 1737 diseases.

The LHGDN is a text-mining derived dataset on GDAs extracted from Entrez Gene’s GeneRIF (21). LHGDN was created based on a GeneRIF version from 31 March 2009, consisting of 414 241 phrases. These phrases were further restricted to the organism *Homo sapiens*, which resulted in a total of 178 004 phrases. We extracted all data from LHGDN and annotated the associations as ‘Biomarker’, ‘Genetic Variation’, ‘PostTranslational Modification’ or ‘Altered Expression’. In total, LHGDN provided 34 487 distinct GDAs for 1846 diseases and 6136 genes.

BeFree: We extracted GDAs from MEDLINE abstracts using the BeFree system. BeFree is composed of a Biomedical Named Entity Recognition module to detect diseases and genes (44) and a relation extraction module based on Support Vector Machine that exploits morphosyntactic information (14). Befree identifies GDAs from text with state-of-the-art performance (F-score 80.9%) (14).

We obtained document set of 737 712 citations from MEDLINE using the following query: (‘Psychiatry and Psychology Category’ [Mesh] AND ‘genetics’ [Subheading]) OR (‘Diseases Category’ [Mesh] AND ‘genetics’ [Subheading]) AND (hasabstract [text] AND (‘1980’ [PDAT]: ‘2014’ [PDAT]) AND ‘humans’ [MeSH Terms] AND English[lang]).

The documents were processed by BeFree trained on the EU-ADR corpus (45) to identify relationships between genes and diseases. This resulted in 530 347 GDAs between 14 777 genes and 12 650 diseases, which were reported in 355 976 publications. Based on an initial analysis of the data, we developed a decision tree workflow to select the most reliable GDAs based on the number of supporting publications, the overlap with other DisGeNET data and the Impact Factor of the journals (see (14) for more details), obtaining 330 888 GDAs between 13 402 genes and 10 557 diseases in 334 943 articles. In addition, we used SETH (46), a tool to perform named entity recognition of single nucleotide polymorphisms (SNP) on the sentences describing the GDA. The tool also assigns dbSNP identifiers (corresponding to NCBI dbSNP Build 137) to the extracted variants. After this normalization process, >8000 SNPs related to ∼3000 genes and 2600 diseases were found. GDAs obtained from BeFree are assigned the association type ‘GeneticVariation’ if there is at least one SNP mediating the association. The rest of the GDAs are classified as ‘Biomarker’.

#### Data organization

We aggregated the data according to their type and level of curation: CURATED (expert-curated associations obtained from UniProt and CTD human datasets), PREDICTED (containing human GDAs inferred from CTD mouse and CTD rat datasets, and from RGD and MGD data), and ALL.

#### Standardization.

##### Gene vocabulary

For human genes, HGNC symbols and UniProt accession numbers have been converted to NCBI Entrez Gene identifiers using an in-house developed dictionary that cross-references HGNC, UniProt and NCBI-Gene information. To map mouse and rat genes to their human orthologs, we used mapping files provided by RGD and MGD. We only kept GDAs for which a human ortholog of the mouse or rat gene was found.

##### Disease vocabulary

The vocabulary used for diseases in the current release of DisGeNET is the Unified Medical Language System (UMLS) Metathesaurus (2013AA release May 2013 version). The repositories of GDAs use different disease vocabularies: MIM terms for OMIM diseases (used by UniProt, CTD and MGD), MeSH terms (used by CTD, LHGDN and RGD). Disease names in GAD are not normalized. We used UMLS Metathesaurus concept structure to map MIM, MeSH, HDO, HPO and ICD9-CM terms, as well as disease names to UMLS Concept Unique Identifiers (CUIs).

#### Data attributes

We mapped the genes to top-level Reactome pathways and to Panther protein classes. We classify diseases according to UMLS semantic types and MeSH classes. In addition, we annotated diseases to the HPO and to the HDO.

##### DisGeNET association type ontology

To characterize the relationships between genes and diseases, we use the DisGeNET association type ontology that has been modified from (12) and was integrated to the Semantics Science Integrated Ontology (SIO) (47) (Figure 5). The ontology is available at http://www.disgenet.org/ds/DisGeNET/files/GeneDiseaseAssociation.owl

##### DisGeNET score

A score has been implemented to assist in the prioritization and navigation of DisGeNET GDAs. For each GDA a score that ranges from 0 to 1 is computed as follows:

(1) $$S=(W_{\text{UNIPROT}}+W_{\text{CTD human}})+(W_{\text{Mouse}}+W_{\text{Rat}})+(W_{\text{GAD}}+W_{\text{LHGDN}}+W_{\text{BeFree}})$$

where:

$$W_{\text{UNIPROT}}=\{\begin{array}{cc} \text{0.3} & \begin{array}{c} \text{if the association has been }\\ \text{reported in Unipropt} \end{array}\\ \text{0} & \text{otherwise} \end{array}$$

$$W_{\text{CTD human}}\text{=}\{\begin{array}{cc} \text{0.3} & \begin{array}{c} \text{if the association has been }\\ \text{reported in CTD human dataset} \end{array}\\ \text{0} & \text{otherwise} \end{array}$$

$$W_{\text{Rat}}\text{=}\{\begin{array}{cc} \text{0.1} & \begin{array}{c} \text{if the association has been }\\ \text{reported in RGD or CTD rat dataset} \end{array}\\ \text{0} & \text{otherwise} \end{array}$$

$$W_{\text{Mouse}}\text{=}\{\begin{array}{cc} \text{0.1} & \begin{array}{c} \text{if the association has been }\\ \text{reported in MGD or CTD mouse dataset} \end{array}\\ \text{0} & \text{otherwise} \end{array}$$

$$W_{\text{Literature}}\text{=}\{\begin{array}{cc} \text{max} & \text{if }\frac{n_{\text{gd}}\text{×100}}{N_{\text{Literature}}}\text{≥max }\\ \frac{{\text{n}}_{\text{gd}}\text{×100}}{{\text{N}}_{\text{Literature}}} & \text{if }\frac{n_{\text{gd}}\text{×100}}{N_{\text{Literature}}}\text{<max } \end{array}$$

and Literature represents GAD, LHGDN or BeFree, n_gd_ is the number of publications reporting a GDA in the source and N_Literature_ is the total number of publications in the source.

$$\text{max}=\{\begin{array}{cc} 0.08 & \text{if Literature=GAD}\\ 0.06 & \text{if Literature=LHGDN}\vee \text{BeFree} \end{array}$$

#### The web interface

The interface is powered by Onexus (http://www.onexus.org/), a framework that manages the storage, visualization and sharing of biological data. It runs on a MySQL database and an Apache Karaf OSGi runtime, and incorporates visual capabilities such as a Google-like search tool and Wolfram alpha-like reports.

#### DisGeNET RDF

The conversion of DisGeNET as a Linked Dataset has been done using the W3C’s recommended standard Semantic Web technologies RDF, RDFS and OWL. The precise meaning of concepts and relations are defined using common ontologies and vocabularies such as Semantic SIO (47) or the National Cancer Institute Thesaurus (http://ncit.nci.nih.gov/) in order to ease the mapping to concepts from other biomedical RDF datasets. Each gene-disease instance is semantically defined using the DisGeNET association type ontology integrated into SIO and identified by resolvable Internationalized Resource Identifiers (IRIs). These IRIs are composed by a namespace (http://rdf.disgenet.org/) and a DisGeNET identifier. Finally, the RDF dataset has been built according to the Linked Data principles (http://www.w3.org/DesignIssues/LinkedData.html) to ensure open access and the data interoperability. This implies that the DisGeNET RDF resources are linked out to other external resources in the Linking Open Data Cloud such as UniProt and several datasets from the Bio2RDF (48) and Linked Life Data (49) subnetworks, spanning the information on genotype-phenotype associations in the Semantic Web and promoting DisGeNET discoverability. DisGeNET RDF is accompanied by a metadata description using the standard VoID to fully describe the provenance and characteristics of the current version of the dataset.

## Conclusions and perspectives

DisGeNET has evolved into a discovery platform to support studies on the mechanisms underlying human diseases. DisGeNET—whose first release was only accessible as a Cytoscape plugin—currently incorporates several powerful features, which are advantageous with respect to similar resources.

DisGeNET integrates information from varied data sources and data types covering different kinds of associations between genes and diseases. This integration offers several advantages. First, we provide standardized annotations of entities (genes and diseases), and their relationships (by means of an ontology), which favors the organization and analysis of the information. Second, thanks to this integration, DisGeNET clearly outperforms similar repositories, taking into account the number of GDAs. Third, we centralize the knowledge scattered across several databases, some of them covering only specific disease areas, in a single platform. To complement the centralized warehouse approach, we also provide a Linked Open Data version of DisGeNET to support its use in a federated manner in the Semantic Web. Furthermore, the DisGeNET score allows ranking the information, which might be useful, especially for some well-studied genes and diseases, with dozens or even hundreds of associations, enabling the user to have a quick glimpse on the relevance of the associations. In addition, it is important to highlight that DisGeNET also includes a data set generated by text mining not available from other sources (the BeFree dataset) which comprises a unique and up-to-date catalog of GDAs. Finally, DisGeNET will maintain a version of the GAD data that has been retired.

Due to the explicit representation of the evidence of GDAs, the user can choose which type of information to use for navigation: one can either explore known and well-established associations, or browse information on animal models extrapolated to human genes. The search can also be expanded and complemented by exploring the associations extracted from the biomedical literature, which are not yet collated by the curated resources. The integration of data from curated resources with text-mined data allows incorporating information from the most recent published scientific studies (14).

DisGeNET provides flexibility in the vocabularies employed to identify both genes and diseases. This allows researchers to query the discovery platform using HGNC Gene Symbols, NCBI, UniProt, MeSH, OMIM, UMLS CUI identifiers, HDO, ICD9-CM and HPO terms or common disease names.

The DisGeNET discovery platform supports very different user profiles with different levels of technical skills: from bioinformaticians to health care practitioners (Box 1). Remarkably, DisGeNET can be accessed and used with a variety of analysis tools offering flexibility in its use and opening the possibility to tackle different applications (Box 2).

**Box 2. Examples of the questions that can be answered using DisGeNET**

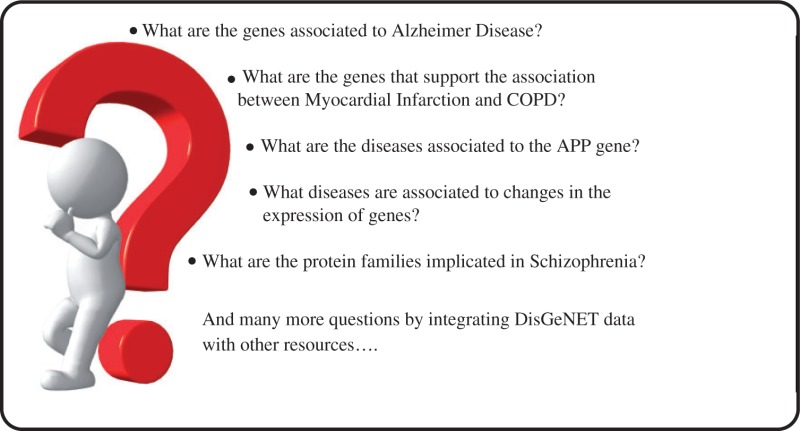

Finally, the DisGeNET content is made available for downloading and sharing under the Open Database License. 

DisGeNET content is also integrated in the Semantic Web as an RDF-Linked Dataset. This allows leveraging from the web of Linked Data to address complex questions in translational research. For all these reasons, DisGeNET constitutes a tool of choice for different types of users interested in the molecular basis of human diseases.

## Supplementary Data

Supplementary data are available at *Database* Online.

## Funding

This work was supported by Instituto de Salud Carlos III-Fondo Europeo de Desarrollo Regional (CP10/00524 and PI13/00082), the Innovative Medicines Initiative Joint Undertaking (115002 (eTOX), 115191 [Open PHACTS]), resources of which are composed of financial contribution from the European Union’s Seventh Framework Programme [FP7/2007-2013] and EFPIA companies’ in kind contribution. The Research Programme on Biomedical Informatics (GRIB) is a node of the Spanish National Institute of Bioinformatics (INB). Funding for open access charge: Instituto de Salud Carlos III-Fondo Europeo de Desarrollo Regional (PI13/00082).

*Conflict of interest*. None declared.

## Supplementary Material

Supplementary Data
