# Supplementary material for: DisGeNET: a discovery platform for the dynamical exploration of human diseases and their genes
Source: Database (Oxford). 2015 Apr 15;2015:bav028. doi: 10.1093/database/bav028 (PMC4397996; doi:10.1093/database/bav028)
Supplement: Supplementary Data [file supp_2015_bav028_index.html]

DisGeNET: a discovery platform for the dynamical exploration of human diseases and their genes — Supplementary Data 

# DisGeNET: a discovery platform for the dynamical exploration of human diseases and their genes

## Supplementary Data

files

**Files in this Data Supplement:**

- Supplementary Data - docx file
- Supplementary Data - docx file
