# Supplementary material for: DisGeNET: a discovery platform for the dynamical exploration of human diseases and their genes
Source: Database (Oxford). 2015 Apr 15;2015:bav028. doi: 10.1093/database/bav028 (PMC4397996; doi:10.1093/database/bav028)
Supplement: Supplementary Data [file supp_bav028_disgenet_2014_final-suppl.docx]

Supplementary material

Table S1

| Disease Terminology | Number of terms in DisGeNET | Coverage in DisGeNET (%) |
| --- | --- | --- |
| **MeSH** | 5134 | 39 |
| **OMIM** | 3932 | 30 |
| **HDO** | 2943 | 24 |
| **HPO** | 1279 | 9 |
| **ICD9-CM** | 2077 | 13 |

Table S2

| Data source | **Use** | **URL** | **Version or download date** |
| --- | --- | --- | --- |
| **CTD** | Gene-disease associations source | http://ctdbase.org/reports/CTD_genes_diseases.tsv.gz | version July 8, 2013, accessed on July 24, 2013 |
| **GAD** | Gene-disease associations source | http://geneticassociationdb.nih.gov/cgi-bin/download.cgi | July 6, 2013 |
| **UniProt** | Gene-disease associations source | http://www.UniProt.org/docs/humsavar, version 2013_08 | July 24, 2013 |
|  | Mapping gene identifiers | ftp://ftp.uniprot.org/pub/databases/uniprot/current_release/knowledgebase/ idmapping/by_organism/HUMAN_9606_idmapping.dat.gz | February 21, 2014 |
| **NCBI-Gene** | Mapping gene identifiers | ftp://ftp.ncbi.nih.gov/gene/DATA/gene_info.gz. | February 21, 2014 |
| **HGNC** | Mapping gene identifiers | http://www.genenames.org/cgi-bin/hgnc_downloads. | February 21, 2014 |
| **RGD** | Gene-disease associations source | ftp://rgd.mcw.edu/pub/data_release/annotated_rgd_objects_by_ontology/with_terms/rattus_terms_rdo | July 29, 2013 |
|  | Rat to human orthology mapping | ftp://rgd.mcw.edu/pub/data_release/RGD_ORTHOLOGS.txt | August 26, 2013 |
| **MGD** | Gene-disease associations source | ftp://ftp.informatics.jax.org/pub/reports/MGI_OMIM.rpt | July 12, 2013 |
|  | Mouse to human orthology mapping | ftp://ftp.informatics.jax.org/pub/reports/HOM_MouseHumanSequence.rpt | August 26, 2013 |
| **Reactome Project** | Mapping genes to pathways | <http://www.reactome.org/download/current/uniprot_2_pathways.stid.txt> | August 2, 2013 |
| **Panther** | Mapping genes to protein class | ftp://ftp.pantherdb.org/sequence_classifications/current_release/PANTHER_Sequence_Classification_files/PTHR9.0_human | 9.0 release, downloaded on April 7, 2014 |
| **HPO** | Mapping disease identifiers | <http://compbio.charite.de/hudson/job/hpo/> | April 30, 2014 |
| **HDO** | Mapping disease identifiers | <http://www.obofoundry.org/cgi-bin/detail.cgi?id=disease_ontology> | April 30, 2014 |
| **UMLS** | Mapping disease identifiers | UMLS Full Release Files <http://www.nlm.nih.gov/research/umls/licensedcontent/umlsknowledgesources.html> | 2013AA release May 2013 version |
| **MeSH®** | Mapping disease identifiers | <http://www.nlm.nih.gov/mesh/filelist.html> | 2013 release |
