# Supplementary material for: DisGeNET: a discovery platform for the dynamical exploration of human diseases and their genes
Source: Database (Oxford). 2015 Apr 15;2015:bav028. doi: 10.1093/database/bav028 (PMC4397996; doi:10.1093/database/bav028)
Supplement: Supplementary Data [file supp_bav028_New_Microsoft_Office_Word_Document.docx]

**Supplementary Material**

**Table S1:** DisGeNET coverage of diseases with MeSH, OMIM, Human Disease Ontology (HDO), Human Phenotype Ontology (HPO), and ICD9-CM vocabularies. Note that the annotations with HDO, HPO and ICD9-CM are only available in the RDF version of the database.

**Table S2:** Files used in DisGeNET, with their version and download date
